# Double Burden of Malnutrition: Examining the Growth Profile and Coexistence of Undernutrition, Overweight, and Obesity among School-Aged Children and Adolescents in Urban and Rural Counties in Henan Province, China

**DOI:** 10.1155/2020/2962138

**Published:** 2020-02-20

**Authors:** Shengsheng Zhou, Bing Ye, Pengyu Fu, Shan Li, Pu Yuan, Li Yang, Xuan Zhan, Feng Chao, Shufang Zhang, Min Qi Wang, Alice Yan

**Affiliations:** ^1^Public Health Institute, Henan Center Disease Control and Prevention, No. 105, Nongye South Road, Zhengdong New District, Zhengzhou, Henan Province, China; ^2^School of Public Health, University of Maryland College Park, College Park, MD 20742, USA; ^3^Joseph J. Zilber School of Public Health, University of Wisconsin Milwaukee, Milwaukee, WI 53201, USA

## Abstract

**Objective:**

To examine the gender, age, and region of residence in the anthropometric and nutritional profiles of children and adolescents aged 6–18 in Henan Province, China's third most populous province.

**Design:**

This cross-sectional study of the China National Nutrition and Health survey (2010–2013) used a multistage cluster sampling technique. The sample included Chinese schoolchildren and adolescents aged 6 to 18 years (1,660 boys and 1,561 girls). Multiple logistic regression models were used to estimate the associations between sociodemographic correlates and overweight or obesity and stunting. *Setting*. Nine districts/counties in Henan Province. *Participants*. 3,221 subjects completed the questionnaire. Sociodemographic information was obtained. Body weight and height were measured.

**Results:**

There were statistically significant regional differences in average height and weight for boys in all age groups. Girls followed the same trends except for height when 15–18 years old. The urban-rural residence differences were found in relation to prevalence of stunting and weight status. Subjects in poor rural areas (15.43%) and ordinary rural areas (15.34%) had higher rates of stunting compared to their urban counterparts. Prevalence of overweight or obesity was highest in big city areas (15.71%) and lowest in ordinary rural areas (6.37%). Being a boy (OR = 1.69, 95% CI = 1.314–2.143), living in a big city (OR = 2.10, 95% CI = 1.431–3.073), or in a small-medium city (OR = 2.28, CI = 1.606–3.247), or being in a younger age group was associated with being overweight or obese. In addition, being a boy, living in a big city, or in a small-medium city, or being younger in age meant they were less likely to be stunted.

**Conclusions:**

A substantial dual burden of malnutrition among children and adolescents in Henan Province was revealed. The urban-rural differences in nutritional status were found. Stunting was more prevalent in rural areas than in urban. In contrast, while the rising problem of childhood and adolescent obesity still exists in the big city, we also found a great spike in obesity in small-medium cities. Evidence also indicated that boys were more likely to be overweight or obese. Our findings suggest that nutrition education, as well as environmental and policy interventions, is needed to target specific geographic regions.

## 1. Introduction

The rapid economic development and urbanization in low- and middle-income countries has led to a transition from a predominance of undernutrition to a phenomenon known as the coexistence of “dual burden” of over- and undernutrition [1–3]. As the largest developing country and the second-largest economy in the world, China is no exception. One of the most recent national studies reported that, in China, 42% of adults and about one-fifth of all children are overweight or obese [4]. Although changes to dietary patterns and rising obesity in Chinese adolescents and children have been reported in the nationally representative China Health and Nutrition Survey (CHNS) [5, 6], malnutrition remains a threat that may hold back a generation of Chinese in certain geographic areas [7]. For instance, while China's dynamic urban population thrives, much of rural China is mired in poverty [8]. Trends in inequalities for undernutrition, including stunted growth and underweight, remain considerable among children and adolescents in poor regions and rural areas in China [2, 7, 9]. In China, the most common cause of malnutrition among children is inadequate food intake, such as unhealthy dietary habits (e.g., consuming food or drinks high in fat, salt, and/or sugar) in urban students. For rural students, the major problem is not lack of calories but rather lack of nutrient-rich food due to poor economic development in rural areas [7].

Both undernutrition- and overnutrition-related overweight or obesity status during childhood are associated with adverse health consequences later in life [10, 11]. Overweight and obesity are not only serious health problems that affect the growth and development of children and adolescents, but they can also cause developmental problems, such as psychological disorders [12], cognitive dysfunction, impaired motor function [13], and altered timing of puberty [14], and may be accompanied by increased risk of multiple comorbidities, including type 2 diabetes, metabolic syndrome in youth and adults, and obesity in adulthood [15, 16]. Similarly, undernutrition is one of the most common causes of morbidity and mortality among children and adolescents throughout the world [17]. The health consequences of a prolonged state of undernutrition include delayed physical growth and brain development and neurological functioning that translates into cognitive impairment [18, 19]. Furthermore, undernutrition is linked to economic outcomes such as decreased earnings potential and productivity in adulthood [18, 20]. The double burden of malnutrition as a complex problem challenges governments and health organizations to tackle opposite ends of the malnutrition spectrum. Therefore, understanding the prevalence and patterns of the problem is crucial for any country's planning and implementation of public health policy.

Henan Province is China's third most populous province with a population of 100 million, where the majority of the population lives in largely rural and less developed areas. In 2009, Henan Province was reported to have a headcount ratio of income poverty of 23.2% [21], while India was last reported at 21.9% in 2011–12 [22]. The headcount ratio here is the proportion of a population that lives on or below the poverty line at $1.90 a day at 2011 international prices. Henan Province is located in the central part of China, occupying an area of 64,000 square miles (159 county-level divisions) with substantial regional differences in economic development [23]. For example, in 2010, the per capita net income of residents in Henan's big urban city was about $1,419, while in ordinary rural and poor rural areas, the income was about $829 and $781, respectively [24]. These disparities are reflective of a typical urban-rural gap. Given Henan Province's sheer size and regional variations, from urban to rural regions and from rich to poor locations, studying changes in anthropomorphic measures could provide worthwhile insights about the double burden of malnutrition for other low-to-middle-income countries.

The aim of the present study is twofold: (1) investigate the gender, age, and regional (urban versus rural) differences in terms of the height, weight, and growth pattern of stunting, and weight status (including underweight, and overweight or obesity) among children and adolescents (6–18 years) using Henan Province's data derived from a nationally representative nutrition and health survey data from 2010 to 2013; (2) examine the associations between potential correlates (age, sex, region of residence, and family income) and overweight or obesity, as well as stunting. Findings about risks or protective factors for growth patterns and weight status can be used to guide the development of a nutrition-related intervention to address undernutrition while preventing obesity.

## 2. Materials and Methods

### 2.1. Study Sample and Settings

A cross-sectional study with the annual China National Nutrition and Health Survey (CHNS) [5, 6, 25, 26] was conducted in Henan Province from September 2010 to January 2014 using a multistage stratified population-based cluster random sampling technique. The China National Bureau of Statistics and the Information Center of the Chinese Center for Disease Control and Prevention (CDC) assisted in designing a sampling frame of the sampling units that include counties (cities, districts) and villages in Henan Province. A total of nine geographic cities/districts/counties were randomly selected based on socioeconomic characteristics. Those nine cities/districts/counties include one big city district, two small-medium cities' districts, four ordinary rural area's counties, and two poor rural area's counties.

We used the nonagricultural population size and/or the per capita income to categorize four geographic regions [27]. The big city in China is defined as the central city of a municipality directly under the central government, nonagricultural population is more than 500,000, and per capita disposable income per year is about $1800. In this study, a provincial capital city with a population of more than 1 million was selected. The small-medium city in China is defined as all the districts and county-level cities except the big city, nonagricultural population between 150,000 and 500,000, and per capita disposable income per year is about $1500. The poor rural counties in China are defined as the lowest-income rural counties identified by the state government for poverty alleviation and development with a primarily agricultural population, and the per capita net income of farmers is less than 60% of the national average. The ordinary rural counties in China are defined as the rural counties besides the poverty-stricken rural with a primarily agricultural population, and per capita disposable income per year is about $1,200 [28, 29].

In each stratum, 450 households were selected using a random sampling method according to the household registration information. For each household selected, every child and/or adolescent member of that household was interviewed. If the sample size of each stratum contained fewer than 20 children and adolescents per age group in any of the age groups (from 6 to 18 years old), then additional subjects were sampled from surrounding schools. Children and adolescents who had physical disabilities were excluded. All adult subjects provided written informed consent to his/her child's participation. Written parent consent forms and schoolchildren assent forms were obtained before collecting data from both school-aged subjects and their patients. Trained interviewers administered the surveys at subjects' homes. All anthropometric measurements were scheduled and performed by trained investigators at the closest community clinic to the subject's home. This study was conducted according to the guidelines laid down in the Declaration of Helsinki and all procedures involving human subjects were approved by the Ethics Committee of the Henan Province Center for Disease Control and Prevention.

### 2.2. Survey Methods

#### 2.2.1. Survey Instrument

The survey instrument used the nationally representative China National Nutrition and Health Survey [5, 6, 25, 26], which includes a household survey, an adult survey, a child survey, and a nutrition survey. Household sociodemographic characteristics, including age, gender, education levels, and annual family income, were collected in the household survey. The questionnaires from the child survey were filled out with the help of parents of children up to 12 years old, whereas 13–18-year-old adolescents filled out the questionnaires independently. Age categories were organized in intervals of 6 to 8 years, 9 to 11 years, and so forth up to 15 to 18 years.

#### 2.2.2. Anthropometric Measurements and Definitions for Stunting, Underweight, Overweight, and Obesity

Anthropometrical measurements were conducted by well-trained health workers who followed a reference protocol recommended by the World Health Organization (WHO) [30]. A total of 3,312 subjects (ages 6–18) were surveyed and measured. The final analytical sample included only 3,221 subjects after the exclusion of incomplete survey forms/questionnaires and nonreturned forms/questionnaires from parents. Body height was measured using a wall-mounted stadiometer (SG210, Nantong Yuejian Fitness Testing Equipment Co., Ltd.) to the nearest 0.1 m, and body weight was measured with the subjects wearing light clothing and no shoes using a lever weighing scale (RGT-14-RT) to the nearest 0.1 kg. With the metric system, the body mass index (BMI) was calculated as the weight in kilograms (kg) divided by meters squared (m^2^).

To define stunting, height-for-age Z-scores were calculated using National Center for Health Statistics (NCHS) reference values with Epi-Info software package (Centers for Disease Control and Prevention (CDC), Atlanta, GA, version 6.03) [31]. Children and adolescents (aged 2–19 years) were classified as stunted/or short status (height-for-age Z-score (HAZ) < 5 percentile) and tall stature (HAZ ≥ 95 percentiles) according to the Growth Standards published by CDC. We also used Epi-Info to calculate BMI percentiles and BMI Z-scores using CDC 2000 Growth Charts as references. Age- and gender-specific cutoff points for BMI from the National Center for Health Statistics (NCHS) references were used to define underweight (BMI < 5 percentiles) and overweight and obesity (BMI ≥ 85 percentiles).

### 2.3. Statistical Analyses

Dependent variables were weight and growth patterns, including indicators for stunting, underweight, and overweight or obesity. Independent variables include an array of sociodemographic variables, such as age, gender, family income, and geographic areas. First, descriptive statistics such as mean and standard deviation were calculated for the continuous variables and frequency distributions were calculated for categorical data. Second, one-way analysis of variance (ANOVA) tests were used to explore regional (big city, small-medium city, ordinary rural, and poor rural areas) mean differences in weight (in kilogram (kg)) and height (in centimeter (cm)) for boys and girls, respectively, stratified by age groups. The Bonferroni correction was used in the post hoc multiple comparisons to correct the inflated type I error rate. Third, chi-square tests were conducted to explore geographic residence differences in stunting and weight status. Finally, the multivariate logistic regression analyses were applied to estimate the associations between potential correlates (age, sex, region of residence, and family income) and overweight or obesity as well as stunting. Odds Ratio and 95% confidence interval were presented. All statistical analyses were performed using the SAS 9.4 for Windows (SAS Institute Inc, Cary, NC), allowing for the complex survey design and nonresponses in both estimates and corresponding standard errors. Survey weights were derived from the 2009 Census and associated administrative data released by the China National Bureau of Statistics. The SAS Proc Survey procedures compute the standard errors for multistage sampling data by including the design factors (i.e., stratum and primary sampling unit). All reported *p* values were based on two-sided tests and statistical significance was set at *p* < 0.05.

## 3. Results

### 3.1. Demographic Characteristics

From 2010 to 2013, a total of 3,221 subjects participated in this study. The sample included 420 (13%) subjects from a big city, 628 (19.5%) subjects from small-medium cities, 1,460 (45.3%) subjects from ordinary rural areas, and 713 (22.1%) subjects from poor rural areas, respectively. Among them, 1,660 (51.5%) were boys and 1,561 (48.5%) were girls. Age group distributions included 26.7% (*n* = 861) schoolers aged 6–8, 25.6% (*n* = 824) schoolers aged 9–11, 25.3% (*n* = 815) schoolers aged 12–14, and 22.4% (*n* = 721) schoolers aged 15–18. Only 3.5% of the sample had self-reported annual per capita income of family more than 3,500 dollars while more than half (59.3%) of the sample reported less than 1,500-dollar annual per capita income (Table 1).

### 3.2. Anthropometric (Height and Weight) Characteristics

Table 2 displayed the means in height (in centimeters (cm)) for boys and girls among four regions of residence and stratified by age groups (6–8, 9–11, 12–14, and 15–18 years old). If the significance was found, the Bonferroni post hoc multiple comparisons among the four regions were presented for both genders.

For boys, the average heights (cm) for age group 6–8 years were 126.15, 126.37, 121.23, and 124.30 in big city, small-medium city, ordinary rural areas, and poor rural areas, respectively. The average height for boys aged 15–18 was 172.91, 169.93, 168.59, and 168.64 in big city, small-medium city, ordinary rural area, and poor rural areas, respectively. There were statistically significant regional differences in average height for boys in all corresponding age groups. In post hoc multiple comparisons, overall, there were no statistical differences in average height for boys between big city and small-medium cities across all age groups. At age 6–8 years, boys in the ordinary rural area were significantly shorter (mean = 121.23) than their counterparts in the other three regions. At age 9–11, boys in big cities (mean = 140.58) and small-medium cities (mean = 142.51) were taller on average than their counterparts in poor rural areas (mean = 134.66). Small-medium city boys at age 9–11 (mean = 142.51) were also taller than their counterparts in ordinary rural areas (mean = 136.98). No statistical difference in average height was observed between boys in two rural areas at age 9–11. At age 12–14, boys in the big city were tallest (mean = 160.59) followed by boys in the ordinary rural area (mean = 155.84), and boys in the poor rural area were the shortest (mean = 152.35).

For girls, the average heights aged 6–8 were 120.52, 125.35, 120.12, and 121.57 in big city, small-medium city, ordinary rural areas, and poor rural areas, respectively. There were statistically significant regional differences in average height for girls in all corresponding age groups except in the age group 15–18 years (*p*=0.06). In post hoc multiple comparisons, overall, there were no statistical differences in average height for girls between big city and small-medium city except for age group 6–8 years. At age 6–8 years, girls in small-medium city were significantly tallest in terms of average height among the four regions. At age 9–11, girls in a big city or a small-medium city were significantly taller than their counterparts in ordinary rural areas and poor rural area. At age 12–14, girls in poor rural areas were significantly shortest.

Table 3 displays the means in weight (kilogram (kg)), for boys and girls among four regions of residence, and stratified by age groups (6–8, 9–11, 12–14, and 15–18 years). In addition, the post hoc multiple comparisons for mean differences among the four regions were presented for both genders.

For boys, the average weights (kg) for age group 6–8 years were 26.74, 26.91, 23.8, and 25.1 in big city, small-medium city, ordinary rural area, and poor rural area, respectively. The average weight for boys aged 15–18 was 62.38, 61.07, 57.7, and 58.7 in big city, small-medium city, ordinary rural area, and poor rural area, respectively. There were statistically significant regional differences in average weight for boys in all corresponding age groups. In post hoc multiple comparisons, overall, there were no statistical differences in average weight for boys between big city and small-medium city across all age groups. Similarly, there were no statistical differences in average weight for boys between ordinary rural areas and poor rural areas across all age groups. For example, at age 6–8 years, boys in both big city (mean = 26.74) and small-medium city (mean = 26.91) were heavier than their counterparts in the ordinary rural area (mean = 23.8). At age 9–11 years, boys in both big city and small-medium city were heavier than their counterparts in ordinary rural area. At age 12–14 years, boys in big city (mean = 51.57) were heavier than their counterparts in two rural areas, and boys in small-medium (mean = 48.04) city were heavier than their counterparts in poor rural area (mean = 43.95). At age 15–18 years, boys in big city (mean = 62.38) and in small-medium city (mean = 61.07) were heavier than their counterparts in ordinary rural area (mean = 57.70), For girls, the average weight (kg) for the age group 6–8 years was 23.6, 25.03, 22.33, and 24.45 in big city, small-medium city, ordinary rural area, and poor rural areas, respectively. The average weight for girls aged 15–18 was 53.23, 53.93, 50.05, and 50.58 in big city, small-medium city, ordinary rural area, and poor rural areas, respectively. There were statistically significant regional differences in average weight for girls in all corresponding age groups. In post hoc multiple comparisons, overall, there were no statistical differences in average weight for girls between big city and small-medium city across all age groups. Similarly, there were no statistical differences in average weight for girls between the ordinary rural area and the poor rural area across all age groups except age group 6–8. At age 6–8, girls in a small-medium city (mean = 25.03) and in a poor rural area (mean = 24.45) were heavier on average than their counterparts in an ordinary rural area (mean = 22.33). At age 9–11, girls in two urban areas were heavier on average than their counterparts in two rural areas. At age 12–14, the statistical difference in mean weight was only observed between girls in a big city and those in a poor rural area. At age 15–18, girls in a small-medium city (mean = 53.93) were heavier than their counterparts in both ordinary rural area (mean = 50.05) and poor rural area (mean = 50.58).

### 3.3. Stunting, Underweight, Overweight, and Obesity

Table 4 illustrates the prevalence of stunting by region of residency and stratified by gender. Overall, from 2010 to 2013, the prevalence of stunting (short stature) in children and adolescents (aged 6–18 years) of Henan Province was 12.95%. There was a significant regional difference in stunting (chi-square = 81.144, *p* < 0.001). Subjects in poor rural areas (15.43%) and ordinary rural areas (15.34%) had higher rates of stunting compared to their urban counterparts. When stratified by gender, similar trends were observed for both boys and girls as rural subjects had higher rates of stunting than their urban counterparts.

Table 5 provides the prevalence of underweight, normal weight, overweight, or obesity by region of residence and stratified by gender. Overall, the prevalence of underweight and overweight or obesity in children and adolescents (aged 6–18 years) of Henan Province was 7.61% and 9.69%, respectively. There were significant regional differences in weight status (chi-square = 68.134, *p* < 0.001). Subjects in the small-medium city had the highest rate (9.24%) and in the big city the lowest rate (4.76%) of underweight. Prevalence of overweight or obesity was highest in the big city (15.71%), followed by the small-medium city (15.29%), poor rural area (7.99%), and ordinary rural area (6.37%). When stratified by gender, boys in a small-medium city had the highest rate of underweight (10.22%) and in the big city the lowest rate (4.9%) of underweight. Similar to boys, girls in the small-medium city had the highest rate (8.25%) and in the big city the lowest rate (4.63%) of underweight. Boys' rate of overweight or obesity was highest in the small-medium city (19.49%), followed by big city (18.63%), ordinary rural area (8.4%), and poor rural area (7.89%). Prevalence of overweight or obesity for girls was highest in the big city (12.96%), followed by small-medium city (11.11%), poor rural area (8.13%), and ordinary rural area (4.23%).

Table 6 shows findings from the multivariate analyses of factors associated with overweight or obesity, as well as stunting. Boys were more likely to be overweight or obese than girls (OR = 1.69, 95% CI = 1.314–2.143). Subjects in a big city were more than two times likely to be overweight or obese (OR = 2.10, 95% CI = 1.431–3.073) compared to subjects in a poor rural area. Likewise, subjects in a small-medium city were 2.28 times likely to be overweight or obese (OR = 2.28, CI = 1.606–3.247) compared to poor rural area subjects. Subjects in age groups 6–8 years (OR = 1.67, CI = 1.164–2.432) and 9–11 years (OR = 1.93, CI = 1.345–2.780) were also more likely to be overweight or obese compared to those aged 15–18 years.

Boys were less likely to be stunted than girls (OR = 0.81, 95% CI = 0.669–0.979). Subjects in the big city were less likely to be stunted (OR = 0.32, 95% CI = 0.221–0.462) compared to subjects in the poor rural area. Likewise, subjects in the small-medium city were less likely to be stunted (OR = 0.44, CI = 0.324–0.605) compared to poor rural area subjects. Subjects in age groups 6–8 years (OR = 0.73, CI = 0.556–0.968) were also less likely to be stunted compared to those aged 15-18 years.

## 4. Discussion

In this cross-sectional study of the China National Nutrition and Health Survey (2010–2013), we first described the anthropometric characteristics (height, weight) of children and adolescents aged 6–18 in Henan Province between 2010 and 2013. Then we examined the gender, age, and regional differences in the anthropometric and nutritional profiles of the study sample. Four regions of residence types that represented different socioeconomic statuses of the population, including big city, small-medium city, ordinary rural, and poor rural, were included in the analyses. Population-based surveys such as the China Health and Nutrition Survey (CHNS) [5, 6] can serve as important sources of information on child health and nutritional status at the population level. Anthropometric measurement in CHNS is a noninvasive and inexpensive way to measure the nutritional status of children and adolescents.

Consistent with the literature, we found that, of school-age children and adolescents (6–18 years) in Henan Province, the nutritional status in urban areas was better than in rural areas, in terms of the height and weight development measured by means of height and weight. It is possibly due to the better socioeconomic development in urban areas than those in rural areas. Growth is an important indicator of the health and nutritional status of a child. Young children are vulnerable because of their high nutritional requirements to support growth and development. Monitoring growth usually includes serial measurements of weight, length, or height for all children, head circumference for infants and toddlers, and interpretation of those measurements relative to the growth of a large sample population of children depicted on a selected growth chart [1]. These measurements help to either confirm a child's healthy growth and development or identify a potential nutritional or health problem before the child's nutritional status or health is seriously compromised.

Findings from our study unveiled the urban-rural differences in nutritional status. The prevalence of overweight or obesity was highest in the big city (15.71%). In contrast, subjects in poor rural areas (15.43%) had the highest rates of stunting. The findings are consistent with the literature as studies suggest overweight or obesity is more prevalent in urban than in rural areas [32–35], trends uncommon across the globe, and unique to the country of China. Surprisingly, subjects in a small-medium city had the highest rate of underweight and second-highest rate of overweight or obesity. This finding may suggest children and adolescents in Henan Province might be at risk for the double burden of underweight and overweight, in particular those in the small-medium city. This is consistent with existing evidence that the dual burden of under- and overnutrition exists among children from birth to 18 years in low- and middle-income countries. The economic development (degree of urbanization [36]) and the transformation of lifestyle in small-medium cities probably play a role in the double burden phenomenon. Although our study did not measure the levels of urbanization, it is possible that children and adolescents from the big and small-medium cities (e.g., more urbanized) may benefit from improved economic status, better access to healthcare, sanitation, and better nutrition when compared to their counterparts from rural areas (e.g., lower urbanized). However, rapid environmental, economic, and social changes that follow urbanization, such as environmental hazards, stressors, and unhealthy diets and lifestyles, may put children in risky situations that might lead to overweight [9, 37]. It should be also noted that not only rural areas but also small-medium cities [38] in China have been experiencing a large scale out-migration to large cities for employment opportunities, which has resulted in a large number of children living apart from their parent(s) in a small-medium city or rural areas [2, 38, 39]. These children, called the left-behind children, are often under the care of their grandparents, who usually have poorer nutritional knowledge and beliefs on healthy eating than parental caregivers in large cities in China [2, 39, 40]. Therefore, those children may be at higher risk of being either overfed (by their grandparents) or malnourished (i.e., undernutrition). Future studies are needed to investigate urban-rural differences and out-migration impacts within the specific sociocultural contexts to shed light on the nutrition paradox in children and adolescents in Henan. The double burden of malnutrition presents a unique challenge to the world. Our findings support the notion that interventions are needed to address not only the rising obesity in urban areas but also the lasting undernutrition problem in rural China.

Our findings also suggest that boys were more likely than girls to be overweight or obese [33–35, 41, 42]. A good understanding of the factors, such as weight management behaviors, weight perceptions, body image, and food parenting behaviors, that contribute to the sex differences in overweight or obesity will shed light to guide intervention efforts [42, 43]. Investments in the early years of childhood can be effective in reducing gender inequality in nutritional health in China.

The study had several limitations. First, the major limitation was the lack of measurements for puberty. Due to cultural limitations, we were unable to collect data related to children's and adolescents' sexual development, which includes the appearance of pubic and underarm hair, the growth and development of sex organs, and, in girls, the start of menstruation. Second, due to the cross-sectional nature of the study, we cannot infer causality. Third, the double burden of malnutrition is characterized by the coexistence of undernutrition along with overweight and obesity, or diet-related noncommunicable diseases, within individuals. This paper did not include indicators about diet-related noncommunicable diseases (such as Type 2 diabetes, heart disease, stroke, and cancer). Lastly, levels of urbanization in four regions of residence were not measured in the current study. Future studies that include comprehensive economic development and rural-to-urban migration indicators are needed to draw solid conclusions about the double burden of the phenomenon of malnutrition in ordinary rural areas in China. Regardless of the limitations, the major strengths of the study include the use of a large and representative sample of Henan children and adolescents, which allowed the stratification by age group, gender, and geographic region. Second, the comprehensive anthropometric measures provided the anthropometric and nutritional profiles of children and adolescents aged 6–18 in Henan Province, China's third most populous province. Few studies have reported the presence of the double burden of malnutrition within a similar population. Our study will make a unique contribution by documenting this phenomenon in Henan Province.

## Figures and Tables

**Table 1:** Characteristics of sampled schoolchildren and adolescents in Henan Province.

		Big city *N* (%)	Small-medium city *N* (%)	Ordinary rural area *N* (%)	Poor rural area *N* (%)	Total *N* (%)
Age groups	6–8	141 (16.4)	135 (15.7)	402 (46.7)	183 (21.3)	861 (26.7)
	9–11	106 (12.9)	175 (21.2)	377 (45.8)	60 (20.2)	824 (25.6)
	12–14	97 (11.9)	167 (20.5)	369 (45.3)	182 (22.3)	815 (25.3)
	15–18	76 (10.5)	151 (20.9)	312 (43.3)	182 (25.2)	721 (22.4)

Sex	Boys	204 (12.3)	313 (18.9)	751 (45.2)	392 (23.7)	1660 (51.5)
	Girls	216 (13.8)	315 (20.2)	710 (45.5)	320 (20.5)	1561 (48.5)

Annual per capita income of family	Low (<$1500)	108 (5.7)	251 (13.2)	1119 (58.6)	431 (22.6)	1909 (59.3)
	Middle ($1500∼3500)	122 (28.1)	110 (25.4)	144 (33.2)	58 (13.4)	434 (13.5)
	High (>$3500)	74 (66.1)	24 (21.4)	14 (12.5)	0 (0)	112 (3.5)
	Refuse to answer	116 (15.1)	243 (31.7)	183 (23.9)	224 (29.2)	766 (23.8)

Total		420 (13)	628 (19.5)	1460 (45.3)	713 (22.1)	3221 (100)

**Table 2:** Means in height (cm) and multiple comparisons for mean differences for boys and girls among four regions of residence, stratified by age groups.

Age groups (yr)	Big city^a^	Small-medium city^b^	Ordinary rural area^c^	Poor rural area^d^	F	P	Pab	Pac	Pad	Pbc	Pbd	Pcd
	Mean (95% CI)	Mean (95% CI)	Mean (95% CI)	Mean (95% CI)								
Boys												
6–8	126.15 (123.67–128.62)	126.37 (124.05–128.69)	121.23 (120.17–122.30)	124.30 (122.84–125.77)	9.952	<0.0001^*∗∗*^	1.000	<0.0001^*∗∗*^	0.900	<0.0001^*∗∗*^	0.689	0.011^*∗*^
9–11	140.58 (138.50–142.66)	142.51 (140.69–144.32)	136.98 (135.68–138.27)	134.66 (132.04–137.28)	11.686	<0.0001^*∗∗*^	1.000	0.108	0.003^*∗*^	<0.0001^*∗∗*^	<0.0001^*∗∗*^	0.357
12–14	160.59 (158.22–162.95)	158.06 (156.18–159.95)	155.84 (154.40–157.29)	152.35 (150.13–154.56)	8.950	<0.0001^*∗∗*^	0.930	0.019^*∗*^	<0.0001^*∗∗*^	0.467	0.001^*∗*^	0.031^*∗*^
15–18	172.91 (170.35–175.47)	169.93 (168.41–171.45)	168.59 (167.53–169.66)	168.64 (167.21–170.06)	4.387	0.005^*∗*^	0.203	0.004^*∗*^	0.010^*∗*^	0.997	1.000	1.000
Girls												
6–8	120.52 (118.36–122.68)	125.35 (123.69–127.02)	120.12 (119.01–121.23)	121.57 (119.60–123.54)	7.637	<0.0001^*∗∗*^	0.002^*∗*^	1.000	1.000	<0.0001^*∗∗*^	0.037^*∗*^	1.000
9–11	142.23 (140.08–144.38)	142.53 (140.54–144.52)	137.56 (136.16–138.97)	137.83 (136.11–139.55)	8.625	<0.0001^*∗∗*^	1.000	0.006^*∗*^	0.043^*∗*^	<0.0001^*∗∗*^	0.006^*∗*^	1.000
12–14	157.66 (155.88–159.45)	155.35 (153.72–156.98)	153.57 (152.47–154.68)	150.01 (146.81–153.1)	7.813	<0.0001^*∗∗*^	1.000	0.060	<0.0001^*∗∗*^	1.000	0.003^*∗*^	0.029^*∗*^
15–18	159.35 (157.25–161.44)	159.61 (157.99–161.23)	158.05 (157.24–158.86)	157.08 (155.39–158.78)	2.495	0.060	n/a	n/a	n/a	n/a	n/a	n/a

*Note*. ^*∗*^*p* < 0.05; ^*∗∗*^*p* < 0.0001. Post hoc multiple comparisons were conducted. Bonferroni adjustment was used for post hoc multiple comparisons. Pab shows *p* value for the test of the mean difference between big city and small-medium city; Pac shows *p* value for the test of the mean difference between big city and ordinary rural; Pad shows *p* value for the test of the mean difference between big city and poor rural; Pbc shows *p* value for the test of the mean difference between small-medium city and ordinary rural; Pbd shows *p* value for the test of the mean difference between small-medium city and poor rural; Pcd shows *p* value for the test of the mean difference between ordinary rural and poor rural.

**Table 3:** Means in weight (kg) and multiple comparisons for mean differences for boys and girls among four regions of residence, stratified by age group.

Age groups (yr)	Big city^a^	Small-medium city^b^	Ordinary rural area^c^	Poor rural area^d^	F	P	Pab	Pac	Pad	Pbc	Pbd	Pcd
	Mean (95% CI)	Mean (95% CI)	Mean (95% CI)	Mean (95% CI)								
Boys												
6–8	26.74 (24.86–28.62)	26.91 (25.17–28.65)	23.80 (22.96–24.65)	25.10 (24.17–26.04)	6.169	<0.0001^*∗∗*^	1.000	0.005^*∗*^	0.521	0.004^*∗*^	0.391	0.449
9–11	36.97 (34.48–39.45)	37.62 (35.41–39.84)	32.95 (31.72–34.18)	31.74 (30.26–33.22)	9.898	<0.0001^*∗∗*^	1.000	0.020^*∗*^	0.003^*∗*^	<0.0001^*∗∗*^	<0.0001^*∗∗*^	1.000
12–14	51.57 (48.21–54.93)	48.04 (45.66–50.42)	45.74 (44.39–47.10)	43.95 (42.29–45.60)	7.253	<0.0001^*∗∗*^	0.288	0.002^*∗*^	<0.0001^*∗∗*^	0.419	0.033^*∗*^	0.915
15–18	62.38 (58.78–65.98)	61.07 (58.29–63.85)	57.70 (56.45–58.96)	58.79 (57.46–60.13)	4.201	0.006^*∗*^	1.000	0.027^*∗*^	0.254	0.045^*∗*^	0.638	1.000
Girls												
6–8	23.60 (22.15–25.05)	25.03 (23.86–26.19)	22.33 (21.79–22.87)	24.45 (22.32–26.58)	5.394	0.001^*∗*^	0.737	0.574	1.000	0.003^*∗*^	1.000	0.040^*∗*^
9–11	35.88 (33.79–37.97)	36.18 (34.18–38.18)	31.91 (30.99–32.83)	32.20 (30.77–33.64)	9.587	<0.0001^*∗∗*^	1.000	0.003^*∗*^	0.033^*∗*^	<0.0001^*∗∗*^	0.004^*∗*^	1.000
12–14	47.32 (45.25–49.38)	46.02 (44.11–47.93)	44.38 (43.10–45.67)	43.27 (41.92–44.61)	3.591	0.014^*∗*^	1.000	0.132	0.023^*∗*^	0.807	0.151	1.000
15–18	53.23 (50.09–56.38)	53.93 (51.76–56.11)	50.05 (49.15–50.96)	50.58 (49.14–52.01)	5.772	0.001^*∗*^	1.0000	0.103	0.377	0.001^*∗*^	0.025^*∗*^	1.000

*Note*. ^*∗*^*p* < 0.05; ^*∗∗*^*p* < 0.0001. Post hoc multiple comparisons were conducted. Bonferroni adjustment was used for post hoc multiple comparisons. Pab shows *p* value for the test of the mean difference between big city and small-medium city; Pac shows *p* value for the test of the mean difference between big city and ordinary rural; Pad shows *p* value for the test of the mean difference between big city and poor rural; Pbc shows *p* value for the test of the mean difference between small-medium city and ordinary rural; Pbd shows *p* value for the test of the mean difference between small-medium city and poor rural; Pcd shows *p* value for the test of the mean difference between ordinary rural and poor rural.

**Table 4:** Prevalence of stunting for total subjects, and for boys and girls, by regions of residence.

	Overall *N* (%)	Big city *N* (%)	Small-medium city *N* (%)	Ordinary rural *N* (%)	Poor rural *N* (%)
Short stature					
Total	417 (12.95)	28 (6.67%)	55 (8.76%)	224 (15.34%)	110 (15.43%)
Female	213 (13.65)	20 (9.26%)	30 (9.52%)	117 (16.48%)	46 (14.38%)
Male	204 (12.29)	8 (3.92%)	25 (7.99%)	107 (14.27%)	64 (16.28%)

Normal					
Total	2711 (84.17)	363 (86.43%)	541 (86.15%)	1214 (83.15%)	593 (83.17%)
Female	1313 (84.11)	183 (84.72%)	275 (87.30%)	585 (82.39%)	270 (84.38%)
Male	1298 (84.22)	180 (88.24%)	266 (84.98%)	629 (83.87%)	323 (82.19%)

Tall stature					
Total	93 (2.89)	29 (6.90%)	32 (5.10%)	22 (1.51%)	10 (1.40%)
Female	35 (2.24)	13 (6.02%)	10 (3.17%)	8 (1.13%)	4 (1.25%)
Male	58 (3.49)	16 (7.84%)	22 (7.03%)	14 (1.87%)	6 (1.53%)

**Table 5:** Prevalence of undernutrition, normal weight, and overweight or obese for total subjects, and for boys and girls, by regions of residence.

	Overall *N* (%)	Big city *N* (%)	Small-medium city *N* (%)	Ordinary rural *N* (%)	Poor rural *N* (%)
Undernutrition					
Total	245 (7.61)	20 (4.76%)	58 (9.24%)	115 (7.88%)	52 (7.29%)
Girls	110 (7.05)	10 (4.63%)	26 (8.25%)	48 (6.76%)	26 (8.13%)
Boys	135 (8.13)	10 (4.90%)	32 (10.22%)	67 (8.93%)	26 (6.62%)

Normal weight					
Total	2664 (82.71)	334 (79.52%)	474 (75.47%)	1252 (85.75%)	604 (84.71%)
Girls	1332 (85.33)	178 (82.41%)	254 (80.63%)	632 (89.01%)	268 (83.75%)
Boys	1332 (80.24)	156 (76.47%)	220 (70.29%)	620 (82.67%)	336 (85.5%)

Overweight or obese					
Total	312 (9.69)	66 (15.71%)	96 (15.29%)	93 (6.37%)	57 (7.99%)
Girls	119 (7.62)	28 (12.96%)	35 (11.11%)	30 (4.23%)	26 (8.13%)
Boys	193 (11.63)	38 (18.63%)	61 (19.49%)	63 (8.40%)	31 (7.89%)

**Table 6:** Multivariate analysis of factors associated with overweight or obese as well as stunting.

	Overweight or obesity			Stunting	
Variables (risk vs. reference)		OR	95% CI	OR	95% CI
Gender	Boys	1.68^*∗∗*^	1.314–2.143	0.81	0.669–0.979
	Girls (reference)				

Regions of residence	Big city	2.10	1.431–3.073	0.32^*∗∗*^	0.221–0.462
	Small-medium city	2.28^*∗∗*^	1.606–3.247	0.44^*∗∗*^	0.324–0.605
	Ordinary rural area	0.79	0.561–1.120	0.99	0.783–1.252
	Poor rural area (reference)				

Age groups (years)	6–8	1.67	1.164–2.432	0.73	0.556–0.968
	9–11	1.93	1.345–2.780	0.92	0.696–1.202
	12–14	1.28	0.868–1.875	1.06	0.810–1.389
	15–18 (reference)				

Family income	High (>$3500)	0.72	0.395–1.311	0.89	0.393–2.562
	Middle ($1500∼3500)	0.88	0.280–2.743	1.05	0.896–3.402
	Low (<$1500) (reference)				

^*∗*^ *p* < 0.05; ^*∗∗*^*p* < 0.0001.

## Data Availability

The data used to support the findings of this study are available from the corresponding author upon request.
